# Association of dexmedetomidine with short-term outcome in patients with cardiogenic shock: a retrospective propensity score-matched cohort study from MIMIC-IV

**DOI:** 10.3389/fphar.2025.1644635

**Published:** 2025-09-15

**Authors:** Linfeng Xie, Jing Chen, Bryan Richard Sasmita, Yuanzhu Li, Suxin Luo, Bi Huang

**Affiliations:** Department of Cardiology, The First Affiliated Hospital of Chongqing Medical University, Chongqing, China

**Keywords:** cardiogenic shock, dexmedetomidine, prognosis, mortality, MIMIC-IV

## Abstract

**Background:**

Dexmedetomidine has been demonstrated to have cardioprotective effects in previous studies, prompting our investigation into its potential to improve survival outcomes in patients with cardiogenic shock (CS).

**Methods:**

This retrospective cohort study analyzed data from the Medical Information Mart for Intensive Care (MIMIC) IV database, focusing on patients with CS. Exposure was defined as intravenous dexmedetomidine administration during intensive care unit (ICU) stay. The primary endpoints included 7-day and 30-day all-cause mortality. External validation was conducted using the eICU 2.0 database.

**Results:**

The pre-matched and propensity score matched cohorts comprised 2,341 and 1,038 patients, respectively. Multivariable Cox regression analysis of the overall cohort revealed that dexmedetomidine administration was significantly associated with reduced risk of both 7-day (hazard ratio (HR) = 0.473, 95% confidence interval (CI): 0.359–0.624, p < 0.001) and 30-day all-cause mortality (HR = 0.606, 95% CI: 0.500–0.735, p < 0.001). This protective association persisted after propensity score matching (PSM) for 7-day (HR = 0.418, 95% CI: 0.317–0.552, p < 0.001) and 30-day mortality (HR = 0.579, 95% CI: 0.475–0.705, p < 0.001). Subgroup analyses demonstrated that patients older than 75 years, those with chronic pulmonary disease, or those with lower systolic blood pressure may not benefit from dexmedetomidine. External validation using 1411 CS patients from the eICU 2.0 database confirmed these findings, with PSM-adjusted analyses showing reduced in-hospital (HR = 0.597; 95% CI: 0.395–0.901; p = 0.014) and in-ICU mortality (HR = 0.425; 95% CI: 0.262–0.689; p < 0.001) among dexmedetomidine treated patients.

**Conclusion:**

Dexmedetomidine administration was associated with reduced risk of 7-day and 30-day all-cause mortality in CS patients, though this protective effect may not be significant in patients over 75 years, those with chronic pulmonary disease, or those with lower systolic blood pressure. Prospective studies are required to validate these findings.

## 1 Introduction

Cardiogenic shock (CS) is a life-threatening systemic hypoperfusion syndrome resulting from severe cardiac dysfunction. Without prompt intervention, it rapidly progresses to irreversible multiorgan failure and carries substantial mortality (van Diepen et al., 2017; Chioncel et al., 2020). Recent data indicate that in-hospital mortality for CS in the United States approaches 37% (Osman et al., 2021). European studies report comparable mortality rates, ranging from 30% to 60% (Chioncel et al., 2020). Current therapeutic interventions with proven efficacy in improving outcomes for CS patients remain limited. Developing novel treatment strategies for CS is therefore imperative (van Diepen et al., 2017).

Dexmedetomidine, a highly selective α2-adrenergic receptor agonist, exhibits sedative, analgesic, and anxiolytic properties (Keating, 2015; Bilotta and Pugliese, 2020; Homberg et al., 2023). This agent is frequently administered to perioperative and intensive care patients (Bilotta and Pugliese, 2020). Recent studies indicate that dexmedetomidine exerts cardioprotective effects (Takahashi et al., 2023) in the setting of cardiac surgery (Ji et al., 2013; Peng et al., 2021; Chen et al., 2022) and acute myocardial infarction (AMI) (Liu et al., 2024) by modulating inflammatory responses (Yang et al., 2017), alleviating microcirculatory dysfunction (Lawrence et al., 1996), attenuating oxidative stress (Han et al., 2019), improving cardiac function and protecting against maladaptive remodeling (Han et al., 2019). These pharmacological effects may collectively improve outcomes in CS patients. However, limited clinical evidence exists on dexmedetomidine’s effects on clinical outcomes in CS patients. Using the Medical Information Mart in Intensive Care-IV (MIMIC-IV) database, this propensity score-matched cohort study investigated dexmedetomidine’s potential therapeutic role in CS, with external validation through an independent dataset.

## 2 Methods

### 2.1 Study design and participants

This retrospective observational cohort study investigated the potential survival benefit of dexmedetomidine in CS patients. We extracted clinical data from MIMIC-IV version 3.0 (Johnson et al., 2023), a publicly accessible database containing 94,458 intensive care unit (ICU) admissions at Beth Israel Deaconess Medical Center between 2008 and 2022. The use of anonymized patient records waived the requirement for individual informed consent. Author L.F.X obtained database access after completing the Collaborative Institutional Training Initiative program (Certification number: 57983166). The study protocol was approved by the Ethics Committee of the First Affiliated Hospital of Chongqing Medical University and complied with the Declaration of Helsinki.

**FIGURE 1:**
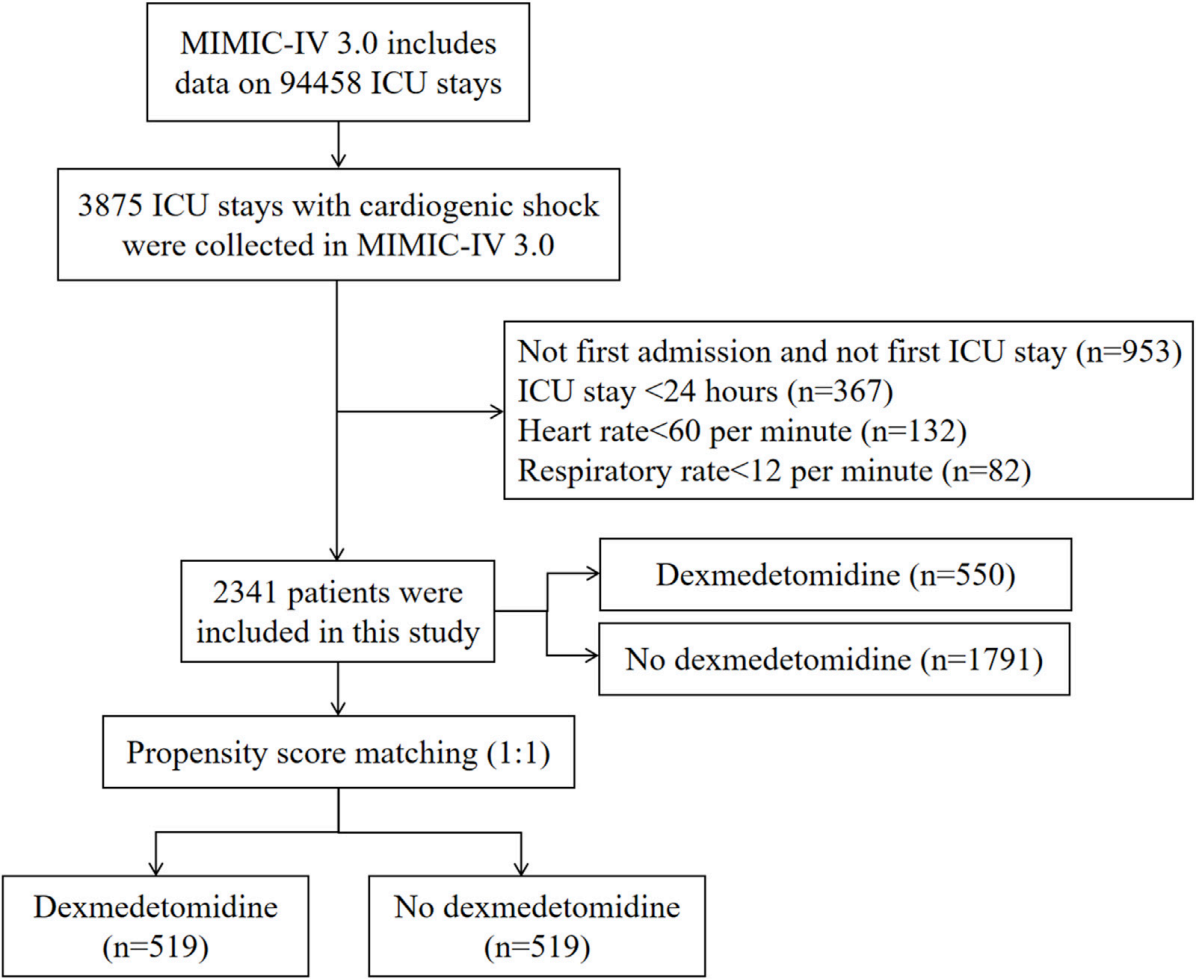
Flowchart of study participants in MIMIC-IV database. ICU, intensive care unit; MIMIC, Medical Information Mart for Intensive Care.

The study enrolled patients diagnosed with CS according to the International Classification of Diseases (9th and 10th Revisions). We excluded individuals with multiple ICU admissions for CS, retaining only data from their first admission, those under 18 years of age, patients with ICU stays shorter than 24 h, patients with heart rate less than 60 per minute, and patients with respiratory rate less than 12 per minute. Figure 1 presents the study flowchart.

### 2.2 Data extraction

L.F.X extracted the data using PostgreSQL (version 13.7.2), Navicat Premium (version 16), and structured query language (SQL). Clinical variables encompassed demographic information, vital signs, initial laboratory parameters post-ICU admission, comorbidities, and treatments. The Sequential Organ Failure Assessment (SOFA) score was also recorded. Dexmedetomidine usage data originated from the prescriptions table in the MIMIC-IV database.

### 2.3 Exposure and study endpoint

Exposure was defined as intravenous dexmedetomidine administration during ICU stay without dosage restrictions. The primary endpoints included 7-day and 30-day all-cause mortality. For those treated with dexmedetomidine, the time of dexmedetomidine administration was taken as the start time of follow-up.

### 2.4 External validation in eICU 2.0 database

The external validation cohort sourced from the publicly available eICU Database (version 2.0), comprised 139,367 patients with 200,859 ICU admissions from 208 U.S. hospitals between 2014 and 2015. We extracted demographic characteristics, laboratory results, treatment records, and mortality outcomes for patients diagnosed with CS.

### 2.5 Statistical analysis

Continuous variables conforming to a normal distribution were expressed as mean ± standard deviation (SD) and compared using independent sample t-tests, whereas non-normally distributed variables were reported as median (25th-75th percentiles) and analyzed with the Mann-Whitney U test. Categorical variables were presented as absolute numbers (percentages) and assessed with either chi-square or Fisher’s exact tests.

To account for potential confounding variables and strengthen the validity of the results, we performed propensity score matching (PSM) following Lonjon et al.'s methodology (Lonjon et al., 2017). Using 1:1 nearest neighbor matching based on propensity scores, we matched patients in the dexmedetomidine and non-dexmedetomidine groups. Standardized mean differences (SMD) below 0.10 confirmed adequate covariate balance before and after PSM (Austin, 2009).

We compared 7-day and 30-day all-cause mortality between the two groups, analyzing cumulative survival probabilities using Kaplan-Meier curves with log-rank tests. Cox proportional hazards models assessed dexmedetomidine’s independent effect on mortality before and after PSM. Three pre-PSM models were constructed: Model 1 remained unadjusted; Model 2 adjusted for demographic and vital signs (age, gender, race, systolic blood pressure, heart rate, respiratory rate); Model 3 adjusted for demographic, vital signs, laboratory values, comorbidities, and interventions (including age, gender, race, systolic blood pressure, heart rate, respiratory rate, temperature, SPO2, hematocrit, hemoglobin, platelet, white blood cell, red blood cell distribution width, anion gap, bicarbonate, blood urea nitrogen, calcium, chlorine, creatinine, glucose, sodium, potassium, SOFA score, AMI, heart failure, cerebrovascular disease, chronic pulmonary disease, liver disease, diabetes, chronic kidney disease, cancer, hypertension, corona virus disease 2019, dobutamine, dopamine, adrenaline, norepinephrine, vasopressin, milrinone, midazolam, diazepam, propofol, fentanyl, invasive ventilation, extracorporeal membrane oxygenation, intraaortic balloon pump, and impella devices). Subgroup analyses evaluated mortality differences across strata defined by age, gender, race, key comorbidities (AMI, heart failure, chronic pulmonary disease, and cerebrovascular disease), and vital signs (heart rate, systolic blood pressure, and respiratory rate). Subgroup analysis based on the timing for dexmedetomidine initiation, duration, and cumulative dose was also conducted. In this study, a 2-tailed p-value of <0.05 was considered statistically significant and all statistical analyses were carried out using SPSS statistical software, version 25.0 (IBM, United States), GraphPad Prism 8.4.3, and R version 4.1.2 (R Foundation).

## 3 Results

### 3.1 The baseline characteristics

The study enrolled 2,341 eligible CS patients (Figure 1), with 550 receiving dexmedetomidine and 1791 not receiving it during ICU admission. The cohort had a mean age of 69.83 years (SD 14.43), and 60.66% (n = 1,420) were male. PSM yielded 1038 CS patients for final analysis.

**FIGURE 2:**
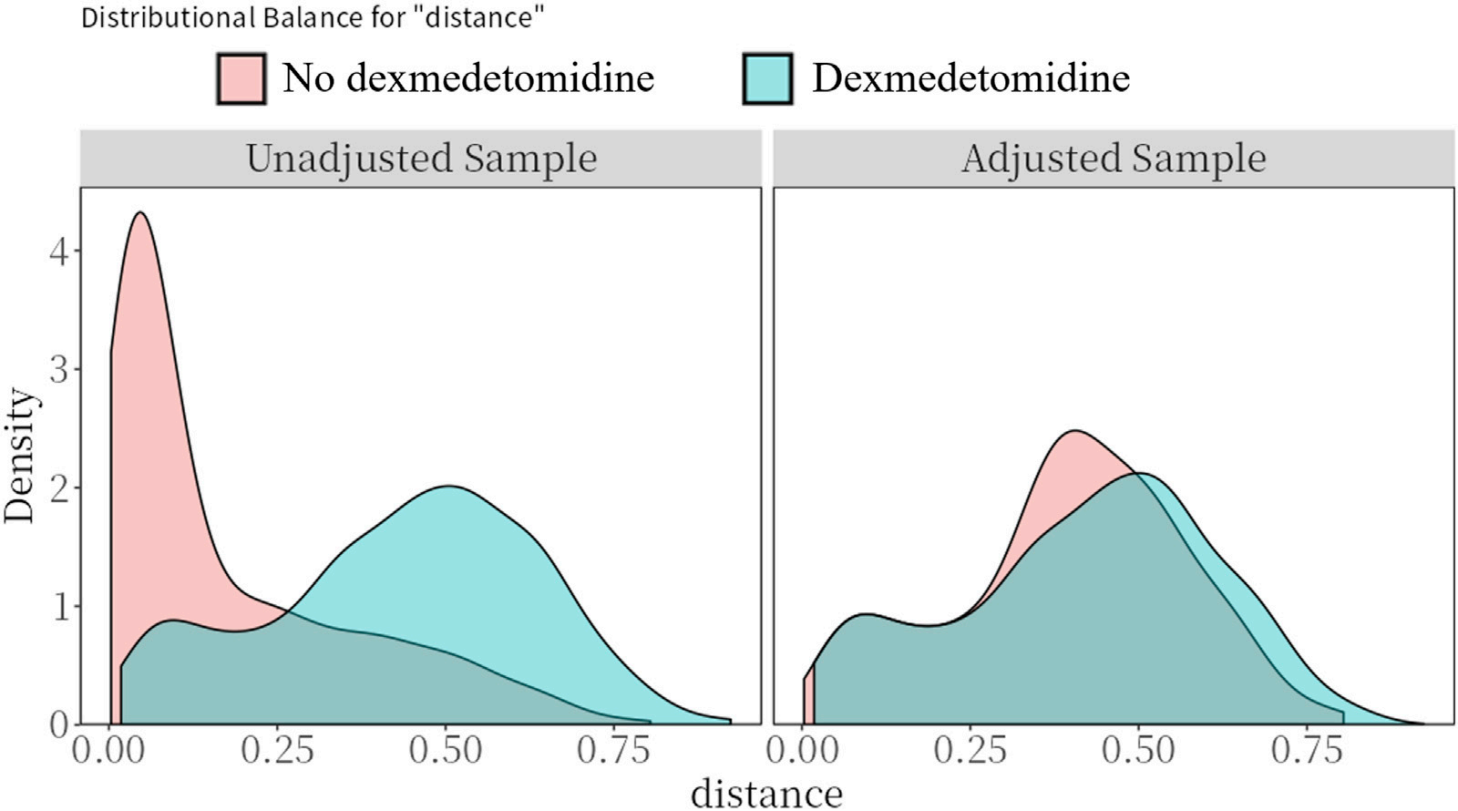
Preference score distributions. Greater overlap indicates that patients in the target and comparator populations are more similar in their likelihood of receiving the target treatment.

Table 1 presents the baseline characteristics of CS patients treated with and without dexmedetomidine. Prior to PSM, significant imbalances existed between the dexmedetomidine and control group in age, gender, systolic blood pressure, prevalence of AMI, chronic kidney disease, cerebrovascular disease, and cancer, along with laboratory values including white blood cell count, anion gap, chlorine, sodium, and blood urea nitrogen. Treatment differences were also observed in the use of vasopressors (dopamine, epinephrine, norepinephrine, vasopressin), other sedative/analgesic medications (midazolam, propofol, fentanyl), invasive ventilation, and intra-aortic balloon pump support (all SMD >0.1). Following PSM, these baseline characteristics achieved balance between the two groups (all SMD<0.1; Table 1; Figure 2).

**TABLE 1:** The baseline characteristics in MIMICIV database.

Variables	Before PSM					After PSM			
	Total (n = 2,341)	No dexmedetomidine (n = 1791)	Dexmedetomidine (n = 550)	P Value	SMD	Total (n = 1,038)	No dexmedetomidine (n = 519)	Dexmedetomidine (n = 519)	SMD
Demographic characteristic									
Age, years, Mean ± SD	69.83 ± 14.43	70.84 ± 14.29	66.54 ± 14.39	<0.001	0.299	67.23 ± 14.47	67.63 ± 14.70	66.84 ± 14.24	0.056
Gender, male, n (%)	1,420 (60.66)	1,061 (59.24)	359 (65.27)	0.011	0.127	662 (63.78)	330 (63.58)	332 (63.97)	0.008
Race, white, n (%)	1,460 (62.37)	1,126 (62.87)	334 (60.73)	0.364	0.044	633 (60.98)	317 (61.08)	316 (60.89)	0.004
Comorbidities									
AMI, n (%)	780 (33.32)	567 (31.66)	213 (38.73)	0.002	0.145	393 (37.86)	194 (37.38)	199 (38.34)	0.020
Heart failure, n (%)	1853 (79.15)	1,434 (80.07)	419 (76.18)	0.050	0.091	783 (75.43)	387 (74.57)	396 (76.30)	0.041
Hypertension, n (%)	536 (22.9)	412 (23.00)	124 (22.55)	0.823	0.011	227 (21.87)	110 (21.19)	117 (22.54)	0.032
Diabetes, n (%)	920 (39.3)	711 (39.70)	209 (38.00)	0.476	0.035	392 (37.76)	198 (38.15)	194 (37.38)	0.016
Cerebrovascular disease, n (%)	307 (13.11)	210 (11.73)	97 (17.64)	<0.001	0.155	172 (16.57)	84 (16.18)	88 (16.96)	0.021
Chronic pulmonary disease, n (%)	683 (29.18)	523 (29.20)	160 (29.09)	0.960	0.002	302 (29.09)	152 (29.29)	150 (28.90)	0.009
Liver disease, n (%)	316 (13.5)	248 (13.85)	68 (12.36)	0.373	0.045	127 (12.24)	60 (11.56)	67 (12.91)	0.040
Chronic kidney disease, n (%)	928 (39.64)	736 (41.09)	192 (34.91)	0.009	0.130	361 (34.78)	181 (34.87)	180 (34.68)	0.004
Cancer, n (%)	213 (9.1)	180 (10.05)	33 (6.00)	0.004	0.171	71 (6.84)	39 (7.51)	32 (6.17)	0.056
COVID-19, n (%)	88 (3.76)	58 (3.24)	30 (5.45)	0.017	0.098	49 (4.72)	23 (4.43)	26 (5.01)	0.026
Vital signs									
SBP, mmHg, Mean ± SD	112.39 ± 22.21	111.46 ± 22.01	115.39 ± 22.59	<0.001	0.174	114.77 ± 22.44	114.28 ± 22.31	115.26 ± 22.57	0.044
Heart rate, per minute, Mean ± SD	94.30 ± 20.97	93.88 ± 20.89	95.66 ± 21.20	0.083	0.084	94.67 ± 21.56	94.24 ± 21.98	95.11 ± 21.16	0.041
Respiratory rate, per minute, Mean ± SD	21.48 ± 6.03	21.39 ± 5.95	21.80 ± 6.26	0.162	0.066	21.60 ± 6.28	21.43 ± 6.31	21.77 ± 6.25	0.054
SPO_2_, %, Mean ± SD	95.88 ± 5.21	95.87 ± 5.14	95.93 ± 5.41	0.787	0.013	95.89 ± 5.77	95.87 ± 6.03	95.91 ± 5.51	0.007
Laboratory test									
Hemoglobin, g/dL, Mean ± SD	11.19 ± 2.54	11.14 ± 2.48	11.33 ± 2.72	0.157	0.068	11.23 ± 2.73	11.17 ± 2.71	11.28 ± 2.75	0.041
Platelet, K/uL, M (Q_1_, Q_3_)	201.00 (145.00, 270.00)	202.00 (146.00, 271.00)	197.00 (142.00, 262.75)	0.316	0.019	201.00 (143.00, 266.00)	202.00 (144.00, 265.00)	201.00 (142.50, 266.00)	0.053
WBC, K/uL, M (Q_1_, Q_3_)	11.90 (8.50, 16.50)	11.80 (8.30, 16.20)	12.55 (9.50, 17.80)	<0.001	0.120	12.60 (9.20, 17.90)	12.50 (9.00, 17.95)	12.60 (9.45, 17.90)	0.005
Anion gap, mEq/L, Mean ± SD	17.46 ± 5.59	17.64 ± 5.47	16.87 ± 5.90	0.005	0.130	17.08 ± 5.93	17.19 ± 5.91	16.97 ± 5.96	0.037
Bicarbonate, mEq/L, Mean ± SD	20.99 ± 5.40	21.08 ± 5.45	20.71 ± 5.23	0.165	0.070	20.60 ± 5.37	20.48 ± 5.48	20.71 ± 5.26	0.042
Calcium, mg/dL, Mean ± SD	8.52 ± 0.98	8.53 ± 0.94	8.47 ± 1.10	0.254	0.050	8.45 ± 1.06	8.43 ± 1.01	8.46 ± 1.10	0.027
Chlorine, mEq/L, Mean ± SD	100.45 ± 7.40	100.18 ± 7.43	101.33 ± 7.27	0.002	0.157	101.38 ± 7.26	101.47 ± 7.18	101.29 ± 7.35	0.024
Potassium, mEq/L, Mean ± SD	4.56 ± 0.99	4.54 ± 0.99	4.62 ± 1.00	0.103	0.079	4.58 ± 1.01	4.54 ± 1.00	4.61 ± 1.01	0.065
Sodium, mEq/L, Mean ± SD	136.65 ± 5.70	136.39 ± 5.70	137.49 ± 5.60	<0.001	0.196	137.49 ± 5.47	137.51 ± 5.31	137.46 ± 5.64	0.009
BUN, mg/dL, M (Q_1_, Q_3_)	31.00 (20.00, 50.00)	33.00 (20.00, 52.00)	26.00 (17.00, 42.00)	<0.001	0.275	27.00 (18.00, 42.00)	27.00 (18.00, 42.00)	27.00 (18.00, 42.00)	0.031
Creatinine, mg/dL, M (Q_1_, Q_3_)	1.50 (1.00, 2.30)	1.50 (1.10, 2.40)	1.40 (1.00, 2.10)	0.001	0.074	1.40 (1.00, 2.08)	1.40 (1.00, 2.00)	1.40 (1.00, 2.10)	0.004
Glucose, mg/dL, M (Q_1_, Q_3_)	147.0 (114.0, 207.0)	146.0 (113.0, 205.8)	152.0 (118.0, 212.0)	0.085	0.005	150.0 (116.0, 213.0)	147.0 (114.0, 214.0)	152.0 (118.0, 207.0)	0.058
Treatment									
Dobutamine, n (%)	514 (21.96)	394 (22.00)	120 (21.82)	0.929	0.004	216 (20.81)	104 (20.04)	112 (21.58)	0.037
Dopamine, n (%)	397 (16.96)	347 (19.37)	50 (9.09)	<0.001	0.358	105 (10.12)	55 (10.60)	50 (9.63)	0.033
Adrenaline, n (%)	441 (18.84)	288 (16.08)	153 (27.82)	<0.001	0.262	287 (27.65)	148 (28.52)	139 (26.78)	0.039
Norepinephrine, n (%)	1,255 (53.61)	898 (50.14)	357 (64.91)	<0.001	0.309	656 (63.2)	328 (63.20)	328 (63.20)	0.000
Vasopressin, n (%)	604 (25.8)	398 (22.22)	206 (37.45)	<0.001	0.315	379 (36.51)	190 (36.61)	189 (36.42)	0.004
Milrinone, n (%)	263 (11.23)	196 (10.94)	67 (12.18)	0.421	0.038	131 (12.62)	68 (13.10)	63 (12.14)	0.029
Midazolam, n (%)	818 (34.94)	572 (31.94)	246 (44.73)	<0.001	0.257	459 (44.22)	226 (43.55)	233 (44.89)	0.027
Diazepam, n (%)	18 (0.77)	10 (0.56)	8 (1.45)	0.068	0.075	9 (0.87)	3 (0.58)	6 (1.16)	0.054
Propofol, n (%)	1,114 (47.59)	650 (36.29)	464 (84.36)	<0.001	1.324	865 (83.33)	432 (83.24)	433 (83.43)	0.005
Fentanyl, n (%)	1,451 (61.98)	949 (52.99)	502 (91.27)	<0.001	1.357	940 (90.56)	469 (90.37)	471 (90.75)	0.013
Invasive ventilation, n (%)	1,411 (60.27)	914 (51.03)	497 (90.36)	<0.001	1.333	939 (90.46)	473 (91.14)	466 (89.79)	0.045
ECMO, n (%)	42 (1.79)	31 (1.73)	11 (2.00)	0.677	0.019	22 (2.12)	11 (2.12)	11 (2.12)	0.000
IABP, n (%)	215 (9.18)	146 (8.15)	69 (12.55)	0.002	0.133	123 (11.85)	57 (10.98)	66 (12.72)	0.052
Impella devices, n (%)	59 (2.52)	38 (2.12)	21 (3.82)	0.026	0.089	39 (3.76)	19 (3.66)	20 (3.85)	0.010
SOFA score, M (Q_1_, Q_3_)	2.00 (0.00, 4.00)	2.00 (1.00, 4.00)	2.00 (0.00, 5.00)	0.362	0.081	2.00 (0.00, 5.00)	2.00 (0.00, 5.00)	2.00 (0.00, 5.00)	0.034

PSM, propensity score matching; AMI, acute myocardial infarction; COVID-19, corona virus disease 2019; SBP, systolic blood pressure; WBC, white blood cell; BUN, blood urea nitrogen; ECMO, extracorporeal membrane oxygenation; IABP, intraaortic balloon pump; SMD, standardized mean differences.

### 3.2 Outcomes

**FIGURE 3:**
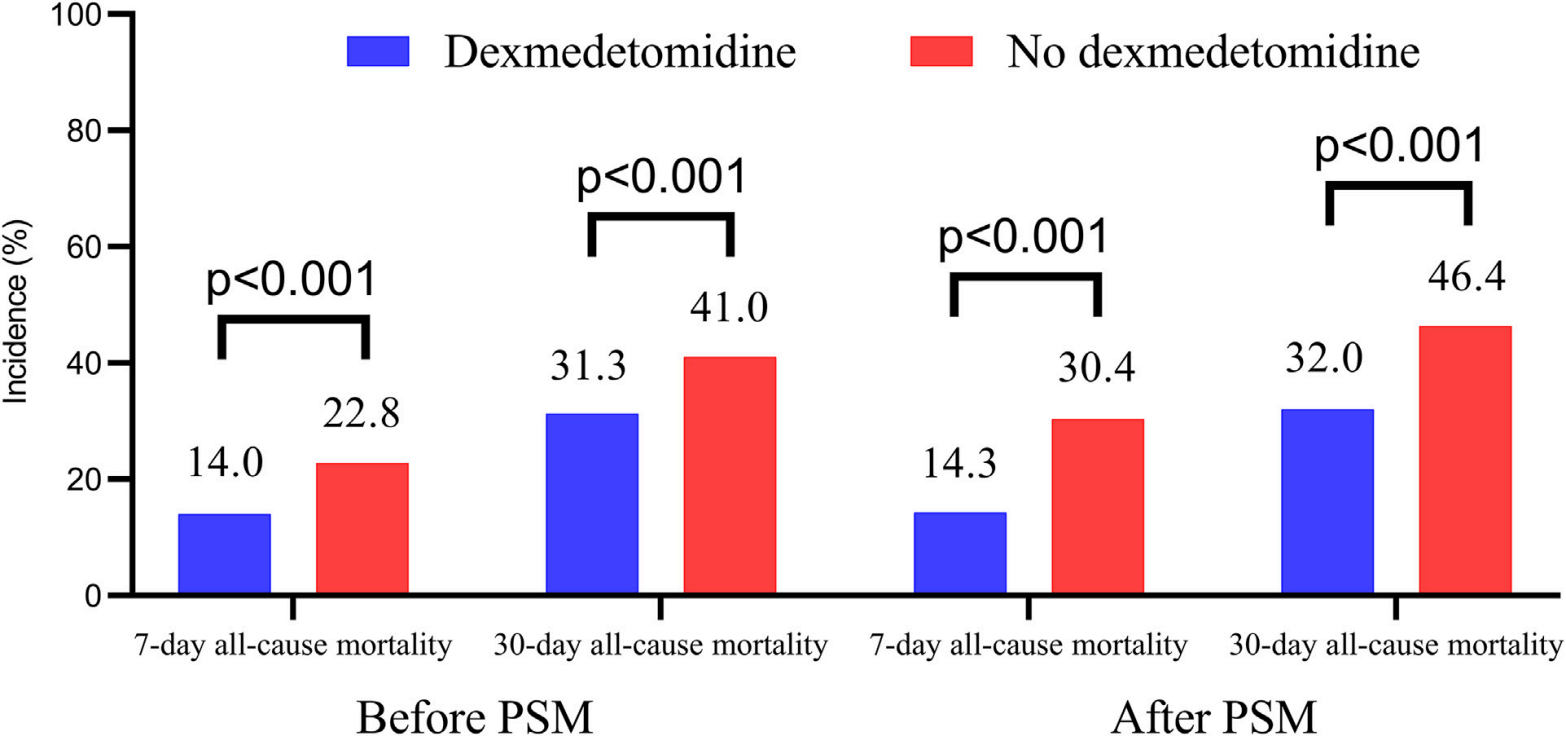
The 7-day and 30-day all-cause mortality in dexmedetomidine and no dexmedetomidine group. PSM, propensity score matching.

Figure 3 presents the 7-day and 30-day all-cause mortality rates. Patients treated with dexmedetomidine exhibited significantly lower 7-day mortality (14.0% vs. 22.8% before PSM, p < 0.001; 14.3% vs. 30.4% after PSM, p < 0.001) and 30-day mortality (31.3% vs. 41.0% before PSM, p < 0.001; 32.0% vs. 46.4% after PSM, p < 0.001).

**FIGURE 4:**
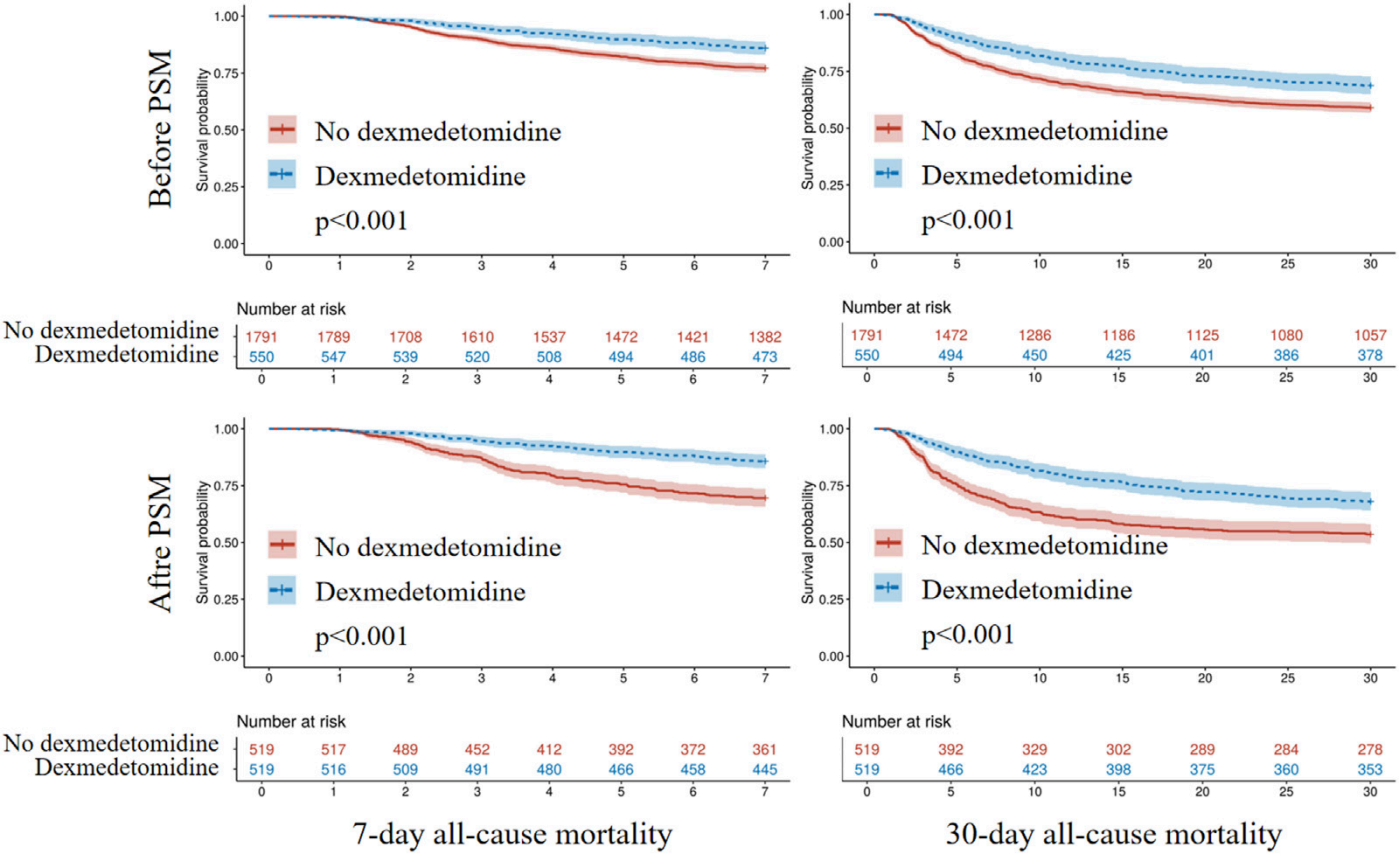
Kaplan-Meier survival analysis curves for all-cause mortality. PSM, propensity score matching.

Kaplan-Meier survival analysis revealed significantly higher 7-day and 30-day survival probabilities in patients receiving dexmedetomidine (Log-rank p < 0.001; Figure 4). Cox regression models adjusted for multiple confounders showed dexmedetomidine was associated with significantly reduced 7-day (HR = 0.473, 95% CI: 0.359–0.624, p < 0.001) and 30-day (HR = 0.606, 95% CI: 0.500–0.735, p < 0.001) all-cause mortality. PSM confirmed this protective effect for both 7-day (HR = 0.418, 95% CI: 0.317–0.552, p < 0.001) and 30-day (HR = 0.579, 95% CI: 0.475–0.705, p < 0.001) all-cause mortality (Table 2).

**TABLE 2:** Association of dexmedetomidine with all-cause mortality in MIMIC-IV database.

Models	7-day all-cause mortality			30-day all-cause mortality		
	HR	95% CI	p	HR	95% CI	p
Before PSM						
Model 1	0.578	0.453, 0.737	<0.001	0.692	0.586, 0.817	<0.001
Model 2	0.617	0.482, 0.789	<0.001	0.762	0.644, 0.902	0.002
Model 3	0.473	0.359, 0.624	<0.001	0.606	0.500, 0.735	<0.001
After PSM	0.418	0.317, 0.552	<0.001	0.579	0.475, 0.705	<0.001

Model 1, unadjusted; Model 2, adjusted by age, gender, race, systolic blood pressure, heart rate, respiratory rate; Model 3, adjusted by age, gender, race, systolic blood pressure, heart rate, respiratory rate, temperature, SPO2, hematocrit, hemoglobin, platelet, white blood cell, red blood cell distribution width, anion gap, bicarbonate, blood urea nitrogen, calcium, chlorine, creatinine, glucose, sodium, potassium, SOFA, score, acute myocardial infarction, heart failure, cerebrovascular disease, chronic pulmonary disease, liver disease, diabetes, chronic kidney disease, cancer, hypertension, corona virus disease 2019, dobutamine, dopamine, adrenaline, norepinephrine, vasopressin, milrinone, midazolam, diazepam, propofol, fentanyl, invasive ventilation, extracorporeal membrane oxygenation, intraaortic balloon pump, and impella devices. HR, hazard ratio; CI, confidence interval; PSM, propensity score matching.

### 3.3 Subgroup analysis

**FIGURE 5:**
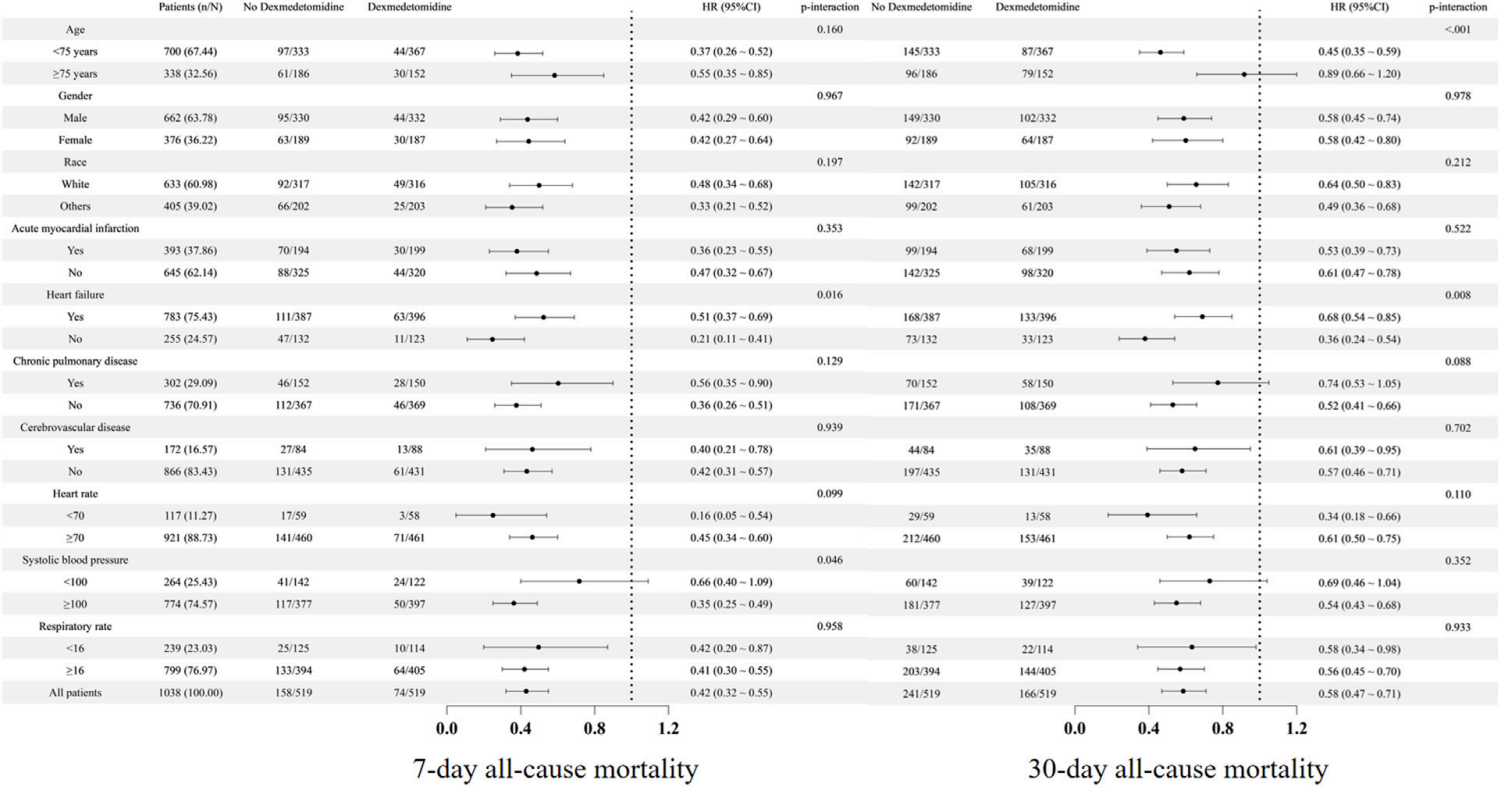
Subgroup analysis. HR, hazard ratio; CI, confidence interval.

To examine the association between dexmedetomidine administration and 7-day or 30-day all-cause mortality across clinical subgroups, we conducted stratified analyses. Dexmedetomidine showed consistent effects on 7-day mortality in most subgroups (p-interaction>0.05). Although the interaction effects were observed in heart failure (heart failure vs. non-heart failure), the effect was only numerically different (Figure 5). Additionally, subgroup analysis suggested an interaction between systolic blood pressure (<100 vs. ≥100 mmHg) and the use of dexmedetomidine in relation to 7-day all-cause mortality (<100 mmHg, HR = 0.66, 95% CI: 0.40–1.09; ≥100 mmHg, HR = 0.35, 95% CI: 0.25–0.49, p-interaction = 0.046). Similarly, its effect on 30-day mortality remained homogeneous across most subgroups (p-interaction>0.05). Although the interaction effects were observed in heart failure (heart failure vs. non-heart failure), the effect was only numerically different (Figure 5). Additionally, subgroup analysis suggested an interaction between age (<75 vs.≥75 years) and the use of dexmedetomidine in relation to 30-day all-cause mortality (<75 years, HR = 0.45, 95% CI: 0.35–0.59; ≥75 years, HR = 0.89, 95% CI: 0.66–1.20, p-interaction<0.001). There was also a trend toward an interaction between the presence of chronic pulmonary disease and the use of dexmedetomidine in relation to 30-day all-cause mortality (with chronic pulmonary disease, HR = 0.74, 95% CI: 0.53–1.05; without chronic pulmonary disease, HR = 0.52, 95% CI: 0.41–0.66, p-interaction = 0.088). The further subgroup analysis based on the timing for dexmedetomidine initiation, duration, and cumulative dose was also conducted (Table 3), which showed that the effect of dexmedetomidine was consistent regardless of timing for dexmedetomidine initiation (within or after 48 h after ICU admission), duration (< or ≥1.25 days), and cumulative dose (< or ≥0.6 mg).

**TABLE 3:** Subgroup analysis based on the duration, dosage, and initiation time of dexmedetomidine in MIMICIV database.

Subgroups	n	7-day all-cause mortality			30-day all-cause mortality		
		HR	95% CI	p	HR	95% CI	p
Duration							
Duration<1.25 days	263/519	0.408	0.284, 0.586	<0.001	0.542	0.419, 0.699	<0.001
Duration≥1.25 days	256/519	0.430	0.302, 0.613	<0.001	0.623	0.488, 0.795	<0.001
Dosage							
Dosage<0.6 mg	371/519	0.418	0.306, 0.570	<0.001	0.562	0.450, 0.702	<0.001
Dosage≥0.6 mg	148/519	0.422	0.267, 0.665	<0.001	0.630	0.465, 0.852	0.003
Initiation time							
Within 48 h after ICU admission	265/519	0.406	0.284, 0.581	<0.001	0.513	0.396, 0.663	<0.001
After 48 h after ICU admission	354/519	0.433	0.303, 0.620	<0.001	0.656	0.515, 0.836	<0.001

HR, hazard ratio; CI, confidence interval; ICU, intensive care unit.

### 3.4 External validation with eICU 2.0 database

The external validation cohort comprised 1411 CS patients from the eICU 2.0 database (Supplementary Figure S1). Baseline characteristics of patients receiving dexmedetomidine versus those without showed balanced covariates after PSM (all SMD<0.1, Supplementary Table S1; Supplementary Figure S2). Multivariate Cox regression prior to PSM (Table 4 Model 3) demonstrated that dexmedetomidine administration significantly reduced both in-hospital (HR = 0.584, 95% CI: 0.400–0.855, p = 0.006) and in-ICU all-cause mortality (HR = 0.451, 95% CI: 0.288–0.706, p < 0.001). Post-PSM analysis (Table 4) confirmed these protective associations, with dexmedetomidine use linked to lower in-hospital (HR = 0.597; 95% CI: 0.395–0.901; p = 0.014) and in-ICU mortality (HR = 0.425; 95% CI: 0.262–0.689; p < 0.001).

**TABLE 4:** Association of dexmedetomidine with all-cause mortality of CS patients in eICU 2.0 database.

Models	In-hospital all-cause mortality			In-ICU all-cause mortality		
	HR	95% CI	p	HR	95% CI	p
Before PSM						
Model 1	0.617	0.442, 0.862	0.005	0.468	0.312, 0.703	<0.001
Model 2	0.597	0.410, 0.870	0.007	0.466	0.299, 0.727	<0.001
Model 3	0.584	0.400, 0.855	0.006	0.451	0.288, 0.706	<0.001
After PSM	0.597	0.395, 0.901	0.014	0.425	0.262, 0.689	<0.001

Model 1, unadjusted; Model 2, adjusted by age, gender, race, systolic blood pressure, heart rate, respiratory rate; Model 3, age, gender, race, systolic blood pressure, heart rate, respiratory rate, hemoglobin, platelet, white blood cell, blood urea nitrogen, calcium, chloride, creatinine, sodium, potassium, hypertension, diabetes, acute myocardial infarction. HR, hazard ratio; CI, confidence interval; PSM, propensity score matching; ICU, intensive care unit.

## 4 Discussion

To our knowledge, this is the first study examining dexmedetomidine’s impact on short-term outcomes in CS patients. Our findings demonstrate that dexmedetomidine administration was associated with significantly reduced risk of 7-day and 30-day all-cause mortality in this population, supporting its clinical use for CS patients if there are indications and no contraindications.

Dexmedetomidine exhibits cardioprotective properties, as demonstrated in prior research. A meta-analysis of 48 trials involving 6,273 cardiac surgery patients revealed that dexmedetomidine shortened intensive care stays by approximately 5 h and reduced tracheal intubation duration by 1.6 h (Poon et al., 2023). Additionally, dexmedetomidine also significantly lowered the relative risk of postoperative delirium (HR = 0.58, 95% CI 0.43–0.78, p = 0.001), atrial fibrillation (HR = 0.76, 95% CI 0.61–0.95, p = 0.015), and short-term mortality (HR = 0.49, 95% CI 0.25–0.97, p = 0.041) (Poon et al., 2023). Another separate PSM analysis of dexmedetomidine in AMI patients revealed significantly lower in-hospital mortality rates among treated individuals (HR = 0.49, 95% CI 0.31–0.77, p = 0.0022) (Liu et al., 2024). These results align with our findings, indicating that dexmedetomidine administration was associated with improved short-term survival outcomes in CS patients.

The mechanisms underlying dexmedetomidine’s survival benefits in CS remain incompletely understood, though several pathways may contribute. First, dexmedetomidine exhibits anti-inflammatory properties by suppressing TNF-α and IL-6 production through inhibition of high mobility group box 1‐toll‐like receptor 4-nuclear factor κB (HMGB1-TLR4-NF-κB) signaling axis (Yang et al., 2017). A meta-analysis of 67 studies involving 4,842 patients demonstrated significantly lower TNF-α and IL-6 levels in patients receiving dexmedetomidine than in controls (Wang et al., 2019). Liu et al. (2024) also demonstrated that 13.7% of dexmedetomidine’s mortality-reducing benefit in AMI patients was mediated through lowered white blood cell counts. Inflammatory responses were significantly activated in CS, leading to impaired cardiac function, circulatory collapse, and adverse clinical outcomes (Shpektor, 2010). Dexmedetomidine demonstrates potential to enhance cardiac function and clinical outcomes in patients with CS by attenuating systemic inflammation. Second, dexmedetomidine significantly reduces myocardial oxygen consumption and cardiac output by decreasing heart rate and blood pressure. Consequently, coronary blood flow decreases physiologically to match the reduced metabolic demand. However, the reduction in heart rate prolongs diastolic perfusion time, which is also beneficial for coronary blood flow. Overall, dexmedetomidine reduces both myocardial oxygen demand and supply, but the reduction in oxygen demand is more pronounced. As a result, dexmedetomidine helps maintain or even improve the myocardial oxygen supply–demand balance (Lee, 2019; Talke et al., 2000; Flacke et al., 1993; Ebert et al., 2000; Weerink et al., 2017). Third, dexmedetomidine markedly redistributes cardiac output, primarily reducing blood flow to less critical tissues such as the skin, spleen, and arteriovenous shunts while enhancing perfusion to vital organs including the heart, brain, and kidneys (Lawrence et al., 1996). Fourth, dexmedetomidine suppressed excessive nicotinamide adenine inucleotide phosphate oxidase-derived oxidative stress, reducing myocardial apoptosis and attenuating subsequent adverse cardiac remodeling (Han et al., 2019). Additionally, dexmedetomidine also attenuates the stress response by suppressing epinephrine, norepinephrine, and cortisol release (Wang et al., 2019). Furthermore, it appears to enhance immune function by elevating natural killer cells, B cells, and CD4^+^ T cell counts while modulating the CD4+:CD8+ and Th1:Th2 ratios (Wang et al., 2019). Given these mechanisms, dexmedetomidine likely confers a survival benefit in CS patients, and our findings confirm its association with reduced short-term mortality.

Although our study suggests that dexmedetomidine administration was associated with improved outcomes in patients with CS, this does not imply that dexmedetomidine should be routinely used in such patients. Instead, the patient’s condition should be comprehensively evaluated and individualized treatment should be administered after assessment by experienced clinicians. Moreover, subgroup analysis revealed an interaction between age and the effect of dexmedetomidine (p < 0.001). In patients older than 75 years, dexmedetomidine use was not significantly associated with prognosis. There was also a trend toward an interaction between chronic lung disease and the effect of dexmedetomidine (p = 0.088); in patients with chronic lung disease, dexmedetomidine use was not significantly associated with prognosis. Moreover, subgroup analysis revealed an interaction between systolic blood pressure and the effect of dexmedetomidine (p = 0.046). In patients with systolic blood pressure less than 100 mmHg, dexmedetomidine use was not significantly associated with prognosis. Older patients often have more comorbidities and markedly reduced dexmedetomidine clearance. Consequently, the higher incidence of adverse reactions, such as hypotension, oversedation, and bradycardia, may offset the potential survival benefits of dexmedetomidine in this population (Weerink et al., 2017). In patients with chronic pulmonary disease, dexmedetomidine’s lack of survival advantage may stem from its respiratory depressant effects (Weerink et al., 2017). In patients with lower systolic blood pressure, dexmedetomidine’s lack of survival advantage may because of the adverse reaction, especially hypotension (Weerink et al., 2017). These findings underscore the need for careful risk-benefit assessment when considering dexmedetomidine in elderly populations, individuals with compromised pulmonary function or lower systolic blood pressure. However, these subgroup findings should be viewed as hypothesis-generating rather than definitive and future prospective studies are needed to confirm these observations and to provide more tailored guidance for clinical practice.

This study has several limitations. First, this is a retrospective observational study, and due to the limitations of its design, it can only establish associations rather than causal relationships. Second, although we employed methods such as propensity score analysis and multivariable regression adjustment, unmeasured confoundings such as physician’s prescribing preferences, subtle clinical indicators, or other unrecorded patient characteristics, etc. Still exist. These unmeasured factors may have influenced both the decision to initiate dexmedetomidine and patient outcomes. Third, while we demonstrated survival benefits of dexmedetomidine in CS patients, its safety profile including hypotension, bradycardia, excessive sedation, and arrhythmias in this population remains unclear due to the limitation of MIMIC-IV database itself, though prior studies demonstrated its safety in cardiac surgery (Poon et al., 2023) and AMI patients (Liu et al., 2024). Fourth, as a sedative, dexmedetomidine is not routinely used in clinical practice and has specific indications. Specifically, dexmedetomidine is often used as an adjunctive agent to facilitate liberation of mechanical ventilation and is an agent to treat delirium in awake patients. Therefore, patients that are improving, awake and able to liberated from mechanical ventilation are more likely to receive dexmedetomidine compared to patients that are not; however, the MIMIC-IV database does not include information on the clinical indications for which the drug was prescribed, and the lack of such data may lead to indication bias. Therefore, caution is needed when interpreting and generalizing the study findings. In addition, the endpoint assessed only all-cause mortality, while cardiovascular mortality, major cardiovascular events, and rehospitalization were unavailable, limiting the comprehensive evaluation of therapeutic efficacy and safety. Therefore, findings from this study needs to be validated by prospective studies, ideally large-scale randomized trials.

## 5 Conclusion

Dexmedetomidine administration was associated with reduced risk of 7-day and 30-day all-cause mortality in CS patients, though this protective effect may not be significant in patients over 75 years or those with chronic pulmonary disease or lower blood pressure. Prospective studies are required to validate these findings.

## Data Availability

The raw data supporting the conclusions of this article will be made available by the authors, without undue reservation.
