# Supplementary material for: Association of dexmedetomidine with short-term outcome in patients with cardiogenic shock: a retrospective propensity score-matched cohort study from MIMIC-IV
Source: Front Pharmacol. 2025 Sep 15;16:1644635. doi: 10.3389/fphar.2025.1644635 (PMC12477159; doi:10.3389/fphar.2025.1644635)
Supplement: Supplementary file 1 [file Supplementaryfile1.docx]

**Table S1** The baseline characteristics in eICU 2.0 database.

|  | Before PSM | | | | | After PSM | | | |
| --- | --- | --- | --- | --- | --- | --- | --- | --- | --- |
|  | Total  (n = 1411) | No dexmedetomidine  (n = 1263) | Dexmedetomidine  (n = 148) | P value | SMD | Total  (n = 474) | No dexmedetomidine  (n = 355) | Dexmedetomidine  (n = 119) | SMD |
| Demographic characteristic |  |  |  |  |  |  |  |  |  |
| Age, years, Mean ± SD | 65.11 ± 13.95 | 65.19 ± 14.01 | 64.52 ± 13.48 | 0.583 | 0.050 | 65.22 ± 13.89 | 65.37 ± 13.90 | 64.77 ± 13.90 | 0.043 |
| Gender, male, n (%) | 887 (62.86) | 786 (62.23) | 101 (68.24) | 0.152 | 0.129 | 305 (64.35) | 226 (63.66) | 79 (66.39) | 0.058 |
| Race, white, n (%) | 1077 (76.33) | 964 (76.33) | 113 (76.35) | 0.995 | 0.001 | 355 (74.89) | 263 (74.08) | 92 (77.31) | 0.077 |
| Comorbidities |  |  |  |  |  |  |  |  |  |
| AMI, n (%) | 524 (37.14) | 464 (36.74) | 60 (40.54) | 0.365 | 0.077 | 199 (41.98) | 151 (42.54) | 48 (40.34) | 0.045 |
| Hypertension, n (%) | 164 (11.62) | 150 (11.88) | 14 (9.46) | 0.385 | 0.083 | 56 (11.81) | 43 (12.11) | 13 (10.92) | 0.038 |
| Diabetes, n (%) | 268 (18.99) | 248 (19.64) | 20 (13.51) | 0.072 | 0.179 | 74 (15.61) | 58 (16.34) | 16 (13.45) | 0.085 |
| Vital signs |  |  |  |  |  |  |  |  |  |
| SBP, mmHg, Mean ± SD | 108.05 ± 26.16 | 107.94 ± 25.88 | 109.03 ± 28.56 | 0.639 | 0.038 | 108.63 ± 26.23 | 108.70 ± 26.18 | 108.41 ± 26.51 | 0.011 |
| Heart rate, per minute, Mean ± SD | 94.93 ± 20.11 | 94.86 ± 20.22 | 95.47 ± 19.19 | 0.727 | 0.032 | 95.44 ± 20.29 | 95.35 ± 20.62 | 95.71 ± 19.36 | 0.019 |
| Respiratory rate, per minute, Mean ± SD | 21.53 ± 7.08 | 21.50 ± 6.58 | 21.86 ± 10.61 | 0.709 | 0.034 | 21.92 ± 7.29 | 22.05 ± 7.34 | 21.50 ± 7.15 | 0.077 |
| Laboratory test |  |  |  |  |  |  |  |  |  |
| Hemoglobin, g/dL, Mean ± SD | 11.84 ± 2.55 | 11.81 ± 2.53 | 12.09 ± 2.77 | 0.213 | 0.100 | 12.17 ± 2.74 | 12.18 ± 2.70 | 12.16 ± 2.87 | 0.007 |
| WBC, K/uL, M (Q₁, Q₃) | 11.51 (8.50, 16.20) | 11.50 (8.45, 16.11) | 12.22 (8.82, 17.70) | 0.103 | 0.089 | 12.34 (9.12, 17.60) | 12.49 (9.15, 17.59) | 12.10 (9.15, 17.40) | 0.086 |
| BUN, mg/dL, M (Q₁, Q₃) | 26.00 (17.00, 42.00) | 26.00 (17.67, 43.00) | 26.00 (16.00, 38.50) | 0.216 | 0.069 | 26.00 (16.00, 39.75) | 26.00 (17.00, 39.00) | 26.00 (16.00, 40.00) | 0.018 |
| Creatinine, mg/dL, M (Q₁, Q₃) | 1.39 (0.99, 2.08) | 1.40 (0.99, 2.10) | 1.30 (1.00, 1.81) | 0.302 | 0.043 | 1.37 (1.00, 2.00) | 1.40 (1.00, 2.10) | 1.28 (1.00, 1.81) | 0.038 |
| Glucose, mg/dL, M (Q₁, Q₃) | 147.0 (114.0, 208.0) | 147.0 (114.0, 206.0) | 146.0 (117.8, 214.8) | 0.491 | 0.032 | 150.0 (115.0, 221.8) | 150.0 (114.0, 220.5) | 149.0 (117.0, 225.0) | 0.045 |

PSM: propensity score matching; AMI: acute myocardial infarction; SBP: systolic blood pressure; WBC: white blood cell; BUN: blood urea nitrogen.


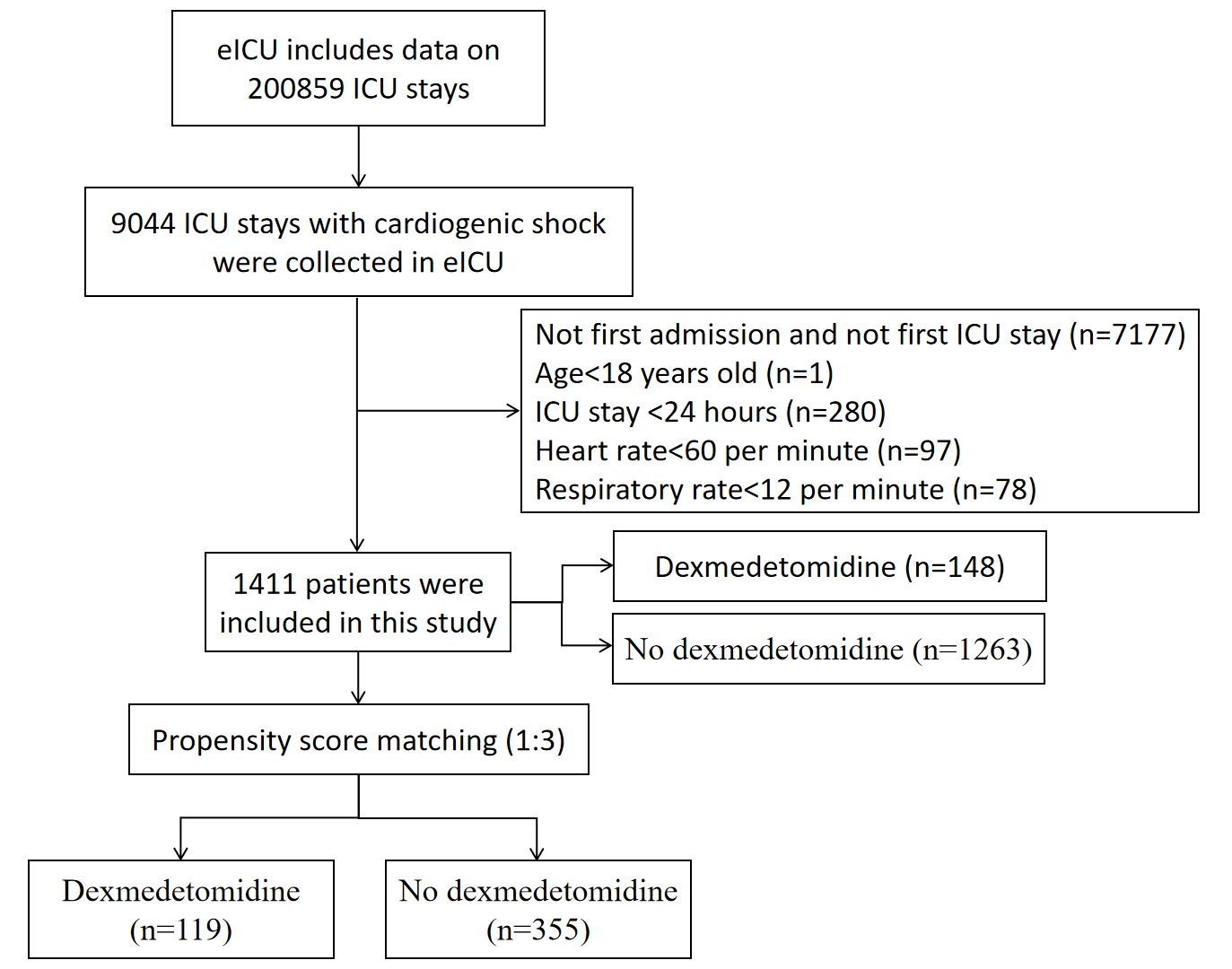


**Figure S1** Flowchart of study participants in eICU 2.0 database. ICU: intensive care unit.

**
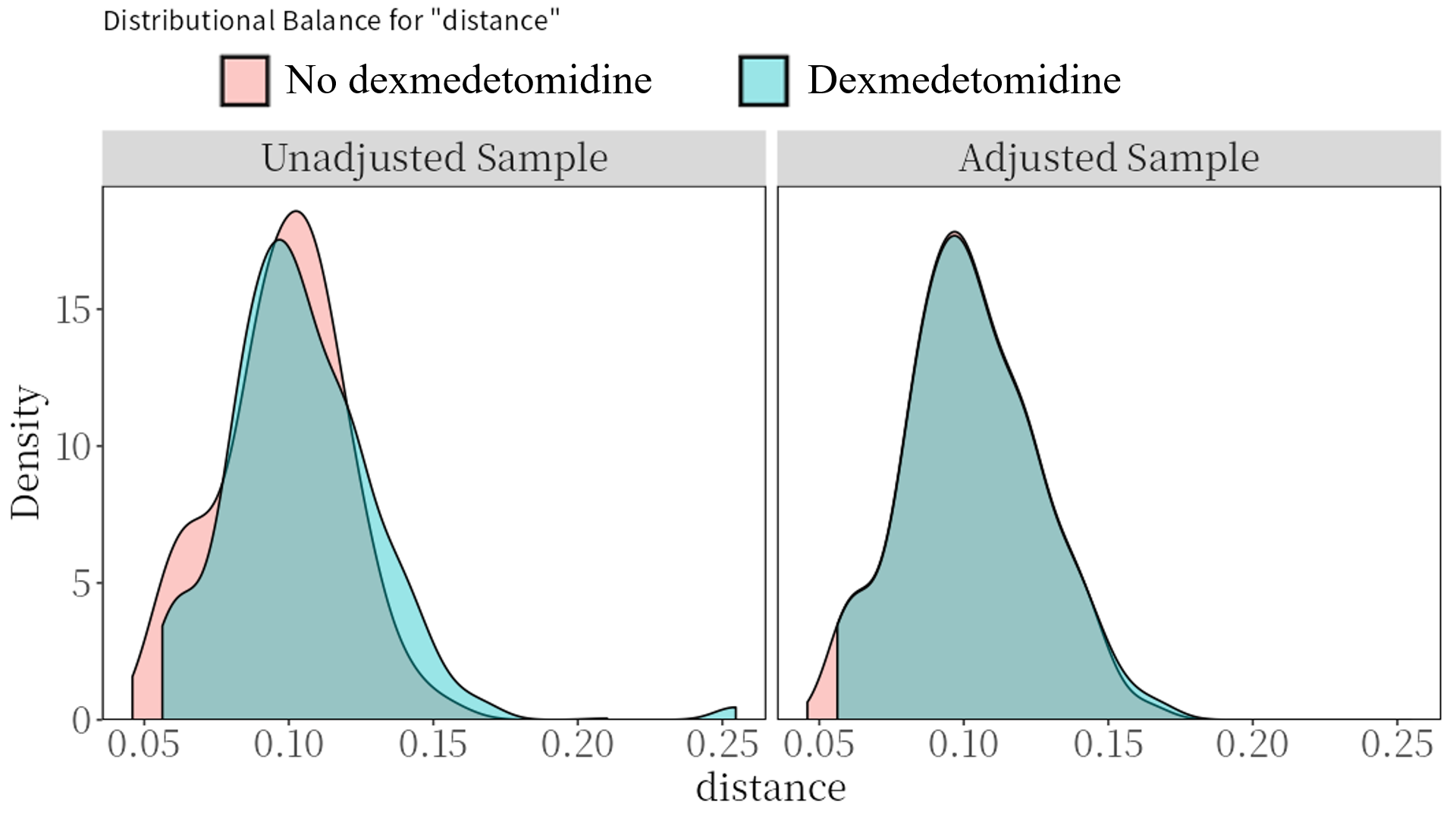
**

**Figure S2** Preference score distributions. Greater overlap indicates that patients in the target and comparator populations are more similar in their likelihood of receiving the target treatment.
