# Supplementary figures and images for: Association of dexmedetomidine with short-term outcome in patients with cardiogenic shock: a retrospective propensity score-matched cohort study from MIMIC-IV
Source: Front Pharmacol. 2025 Sep 15;16:1644635. doi: 10.3389/fphar.2025.1644635 (PMC12477159; doi:10.3389/fphar.2025.1644635)

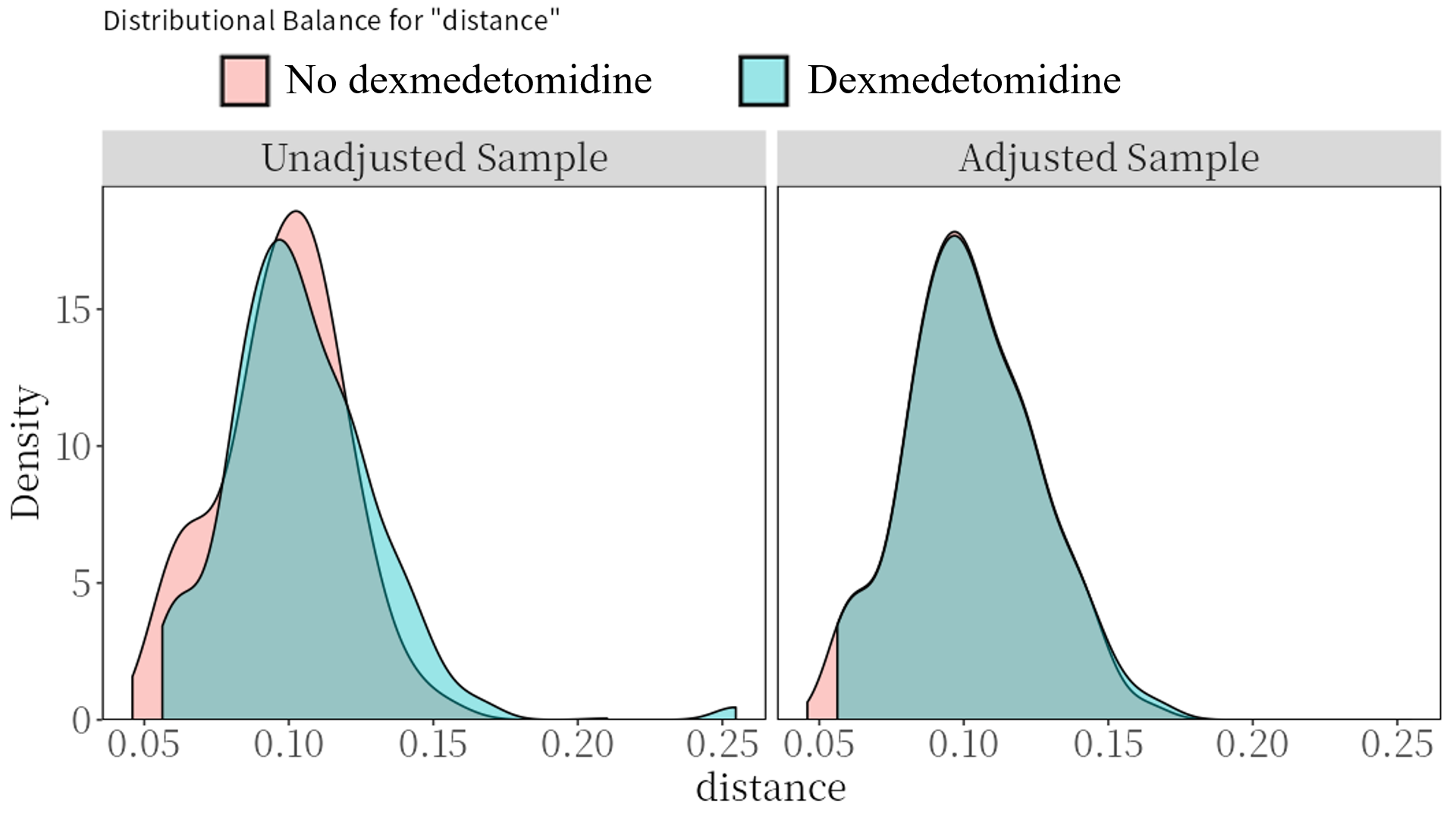

Supplement: Supplementary file 2 [file Image2.tif]

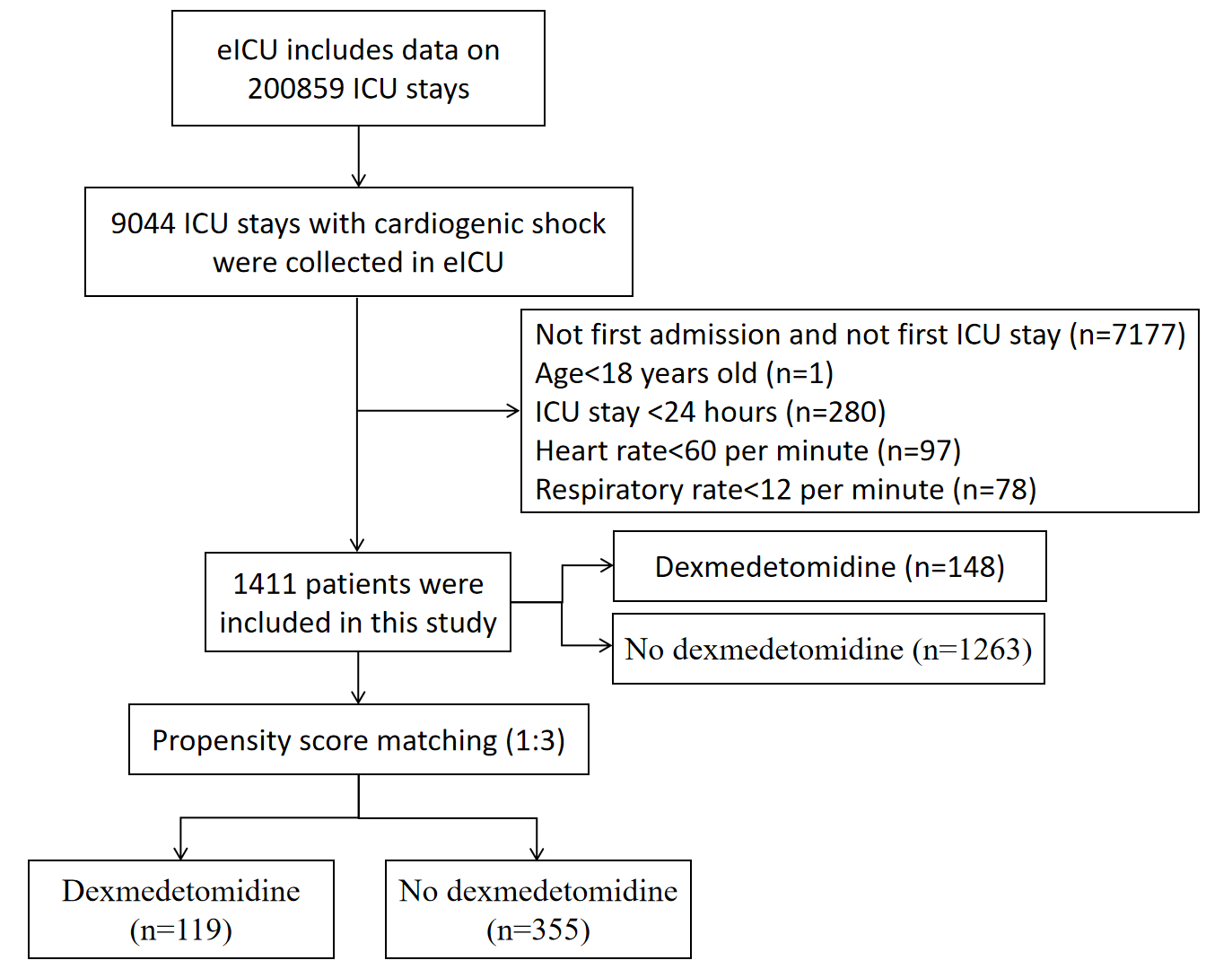

Supplement: Supplementary file 3 [file Image1.tif]
